# A single 1,500 m freestyle at maximal speed decreases cognitive function in athletes

**DOI:** 10.3389/fpsyg.2023.1283585

**Published:** 2023-12-06

**Authors:** Zhijie Lai, Weiwei Huang, Wentao Lin, Xiquan Weng, Yuheng Mao, Guoqin Xu

**Affiliations:** ^1^Department of Graduation, Guangzhou Sport University, Guangzhou, China; ^2^Department of School of Physical Education, Guangzhou College of Commerce, Guangzhou, China; ^3^Department of Physical Education, Guangzhou Sport University, Guangzhou, China; ^4^Department of School of Physical Education, Zhuhai College of Science and Technology, Zhuhai, China; ^5^Department of Sports and Health, Guangzhou Sport University, Guangzhou, China

**Keywords:** 1,500 m freestyle, maximal speed, cognitive function, fNIRS, SGT, TMT, DST

## Abstract

**Introduction:**

Physical exercise can improve cognitive function, and the degree of impact on cognitive function is related to exercise modality, intensity, and duration. However, few studies have been conducted on the effects of competitive sports on cognitive function. The 1,500 m freestyle is the longest pool-based swimming event in the Olympic Games. This study explores the effects of 1,500 m freestyle at maximal speed on athletes’ cognitive function and analyzes the potential mechanism of cognitive function reduction in freestyle at maximal speed from the perspective of hemoglobin oxygenation difference (Hbdiff).

**Methods:**

A total of 13 male university swimmers were required to take part in a 1,500 m freestyle competition, swimming at maximal speed. The relevant indicators, including cognitive function and freestyle at maximal speed, before and after the competition were tested and analyzed. Cognitive function was assessed using the Schulte grid test (SGT), the trail-making test (TMT), and the digit span test (DST). The neurobiological characteristics of cognitive function, such as the prefrontal cortex (PFC), response time (RT), and accuracy rate (ACC), were tested using functional near-infrared spectroscopy (fNIRS).

**Results:**

A significant decrease in scores for SGT, TMT, and digit span test-backward (DST-B) (*p* < 0.01). Oxygenated hemoglobin (Oxy-Hb) concentrations in the right frontopolar area (R-FPA) of brain channels 8 (*p* < 0.01) and 9 (CH8, 9) (*p* < 0.05), the right dorsolateral prefrontal cortex (R-DLPFC) CH10 (*p* < 0.05), and the middle dorsolateral prefrontal cortex (M-DLPFC) CH18 (*p* < 0.01) were significantly altered, and the right area of the brain was activated. The total Oxy-Hb concentrations in the regions of interest (ROIs) of R-FPA, R-DLFPC, and M-DLFPC were changed significantly (*p* < 0.01).

**Discussion:**

The exhaustive performance of a 1,500 m freestyle event resulted in both physical fatigue and a decline in cognitive function. This decline may be attributed to the activation of specific regions of interest, namely the FPA, DLPFC, and M-DLPFC, within the prefrontal cortex (PFC), as well as alterations in functional connectivity.

## 1 Introduction

Cognitive function refers to the brain’s ability to process and understand information, which plays a very important role in learning ability, attention, memory, spatial perception, and problem-solving ability. Cognitive function can be improved through learning and practicing processes, in which exercise is an important factor. Studies have shown that the effect of physical activity on cognitive function depends on the exercise intensity (Weng et al., 2015; Kao et al., 2017; McMorris, 2021). Most studies focused on improving cognitive function through proper physical exercise, and regular aerobic exercise shows benefits in cognitive function improvement (Uysal et al., 2015; Tsai et al., 2018; Amjad et al., 2019; Tomoto et al., 2021). The effects of acute moderate-intensity aerobic exercise on cognitive performance are significantly greater than those of acute low-intensity and high-intensity aerobic exercise (McMorris and Hale, 2012; Ludyga et al., 2016; Devenney et al., 2019; Song and Doris, 2019). Furthermore, cognitive function could be significantly improved both immediately and for a period of time after high-intensity interval training (HIIT) in adolescents, and the benefits of HIIT were better than those of moderate-intensity endurance exercise and lasted longer (Tsukamoto et al., 2016; Coetsee and Terblanche, 2017; Cooper et al., 2018; Kao et al., 2018). The cognitive response in sports is very important for competitive performance. However, far too little attention has been paid to the impact of the conditions of sport on cognitive function, such as intensity and duration.

Exercise could increase the volume of regions such as the PFC and hippocampus, enhance synaptic plasticity, and improve cognitive function and brain health (Ando et al., 2022). Compared with high-intensity intermittent exercise, the effect of moderate-intensity exercise on improving cognitive function is superior to that of information processing speed, which is related to the fact that university students consume a large amount of physical energy during high-intensity exercise (Coetsee and Terblanche, 2017). High levels of arousal lead to premature fatigue and divert the individual’s attention from the current task to feelings of physical discomfort, which could affect behavioral performance and lead to cognitive decline (de la Rosa et al., 2020). Different exercise modalities have different degrees of impact on cognitive function (Brisswalter et al., 1997; Jin et al., 2019; Blumenthal et al., 2020; Broadhouse et al., 2020; Hortobágyi et al., 2021; Pérez-Sousa et al., 2021; Soysal et al., 2021). The well-established benefits of physical activity for cognitive function have been observed with aerobic exercises, the combination of aerobic exercises and strength exercises, and dance movement interventions (Rehfeld et al., 2017; Zhu et al., 2020). There is substantial evidence that physical activity interventions can change the amplitude of fluctuations in the DLPFC and PFC measured by fNIRS and delay the atrophy of brain regions such as the hippocampus (Chen et al., 2016; Tao et al., 2017; Northey et al., 2018). Resistance training has been found to increase the thickness of the PFC, reduce the volume of diseased white matter and white matter atrophy, and improve executive function (Liu-Ambrose et al., 2012; Herold et al., 2019). Participation in both anaerobic resistance group training above the anaerobic threshold intensity and acute high-intensity resistance exercise in middle-aged women improves executive function performance in RT and oral fluency and significantly improves cognitive transitions and refreshment (Chang et al., 2014; Yoon et al., 2017).

Studies have shown that swimming training can improve cognitive function by modulating neuromuscular inflammation-related signaling pathways (Khan et al., 2015). However, the continuum of cognitive effects regulated by swimming and the underlying mechanisms of action have not been fully elucidated. Analyzing the cognitive mechanisms regulating departure reaction time and competition strategies is of great significance in improving athletic performance. The 1,500 m freestyle is the longest pool-based swimming event in the Olympic Games. Changes in the cognitive function of athletes during freestyle at maximal speed greatly affect sports performance. Therefore, we aimed to (1) characterize the impact of 1,500 m freestyle at maximal speed on athletes’ cognitive function and (2) investigate the potential impact of inhibitory control ability in the Stroop task measured by fNIRS and serum serotonin (5-HT) after the 1,500 m freestyle at maximal speed. We hypothesized that cognitive function would decrease after 1,500 m freestyle at maximal speed, and the decrease would be associated with the activated network of brain regions in the right prefrontal cortex.

## 2 Materials and methods

### 2.1 Subjects

A total of 13 male swimmers were recruited from Guangzhou Sport University and participated in the current experiment. All participants were healthy college students who were mostly involved in middle- and long-distance swimming. Inclusion criteria included (1) non-drinker and non-smoker and (2) not fatigued in the past 2 days. All participants were informed of the study procedures and objectives verbally and in writing and signed a written informed consent form. The study was approved by the Ethics Committee of Guangzhou Sports University (Approval No. 2020LCLL-006). The basic parameters of the physical characteristics are shown in Table 1.

**Table 1:** Athlete characteristics.

	Age (y)	Height (cm)	Weight (kg)	BMI (kg/m^2^)	HR (bpm)	Training (y)
*N* = 13	19.15 ± 1.07	180.31 ± 5.04	76.15 ± 9.72	24.25 ± 2.92	58.00 ± 4.76	8.54 ± 1.27

BMI, body mass index; HR, heart rate; Training, years of swimming training.

### 2.2 Experimental program

**Figure 1:**
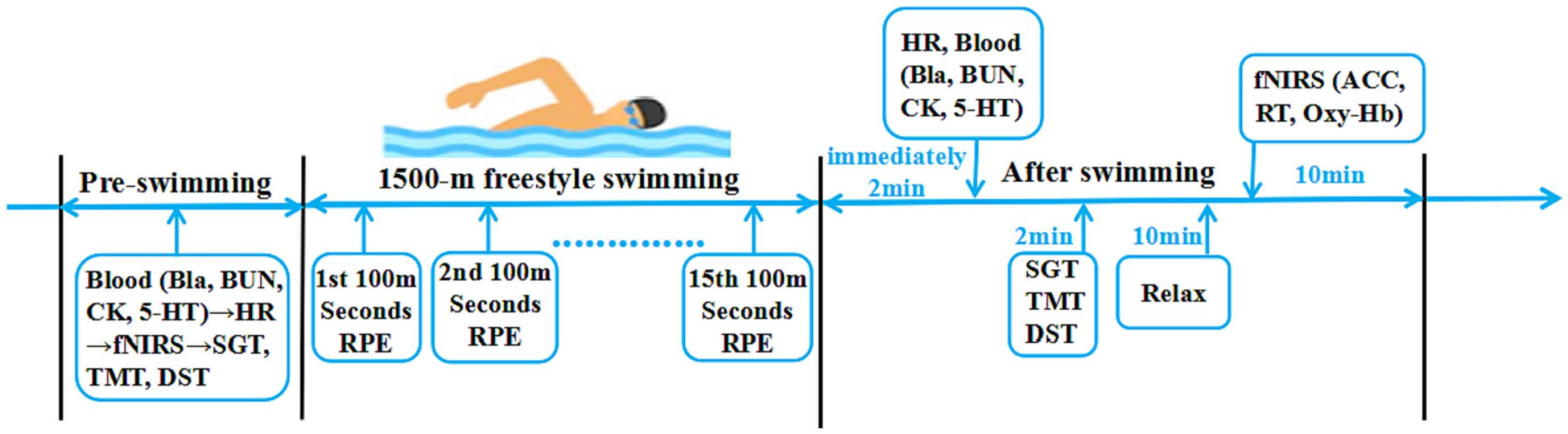
The experimental program. Cognitive function: SGT, Schulte grid test; TMT, trail making test; DST, digit span test; fNIRS functional near-infrared spectroscopy test: ACC, accuracy rate; RT, response time; Oxy-Hb, oxygenated hemoglobin. Physical performance: HR, heart rate; Bla, blood lactic acid; BUN, blood urea; CK, serum creatine kinase; 5-HT, serum serotonin. Effort: RPE, rating of perceived exertion.

On the morning of the swimming session, fasting venous blood samples were collected, and heart rate data were obtained using a heart rate monitor (POLAR RC3, Finland). fNIRS tests and cognitive function assessments were performed using the SGT, DST, and TMT. After reporting a rating of perceived exertion (RPE) value and warm-up, all athletes commenced a 1,500 m freestyle swimming session at the speed of their best previously recorded swimming performance (Matthews et al., 2017). The physical performance of athletes was evaluated by testing the athlete’s RPE value and heart rate (HR) during training. After the swimming session, the RPE scale was recorded to monitor the degree of physical fatigue; venous blood samples were collected again, the second cognitive function assessment was conducted, and then the fNIRS test was conducted (Figure 1).

### 2.3 Physical performance test

The RPE scale was recorded to monitor the degree of physical fatigue (Borg, 1982). We measured the Bla, CK, and BUN to reflect the level of exertion; Bla was tested using an EFK semiautomatic lactic acid analyzer (EFK, Germany). Serum creatine kinase (CK) and blood urea (BUN) were detected using an automatic biochemical analyzer (Chemray-420, Rayto, China). The 5-HT reflected central fatigue, which relates to cognitive function, and 5-HT was tested using ELISA test kits (ABN-KA1894 Serotonin ELISA Kit, USA). The HR was recorded using POLAR RC3 (POLAR, Finland).

### 2.4 Cognitive function assessment

Cognitive function was measured using SGT, DST, and TMT cognitive scales before and after swimming sessions. The SGT, which assesses control ability, a Schulte grid card with 25 squares filled with random Arabic numerals from 1 to 25, requires the athlete to point out their positions in order from 1 to 25 using their fingers while reciting them out loud, and the time taken is the SGT score (Li et al., 2015). The DST, which assesses the attention and cognitive speed, including DST-F (digit span test-forward) and DST-B (digit span test-backward), requires athletes to repeat the sequence of numbers heard in forward and backward directions, and the number of correct numbers is the DST score (Simard and Reekum, 1999). TMT, which assesses attention, a linking scale with 8 numbers and 8 letters in a random order, requires the athlete to link the randomly arranged numbers and letters in alternating order in the fastest time possible, and the time taken is the TMT score (Vickers et al., 1996).

### 2.5 fNIRS test

**Figure 2:**
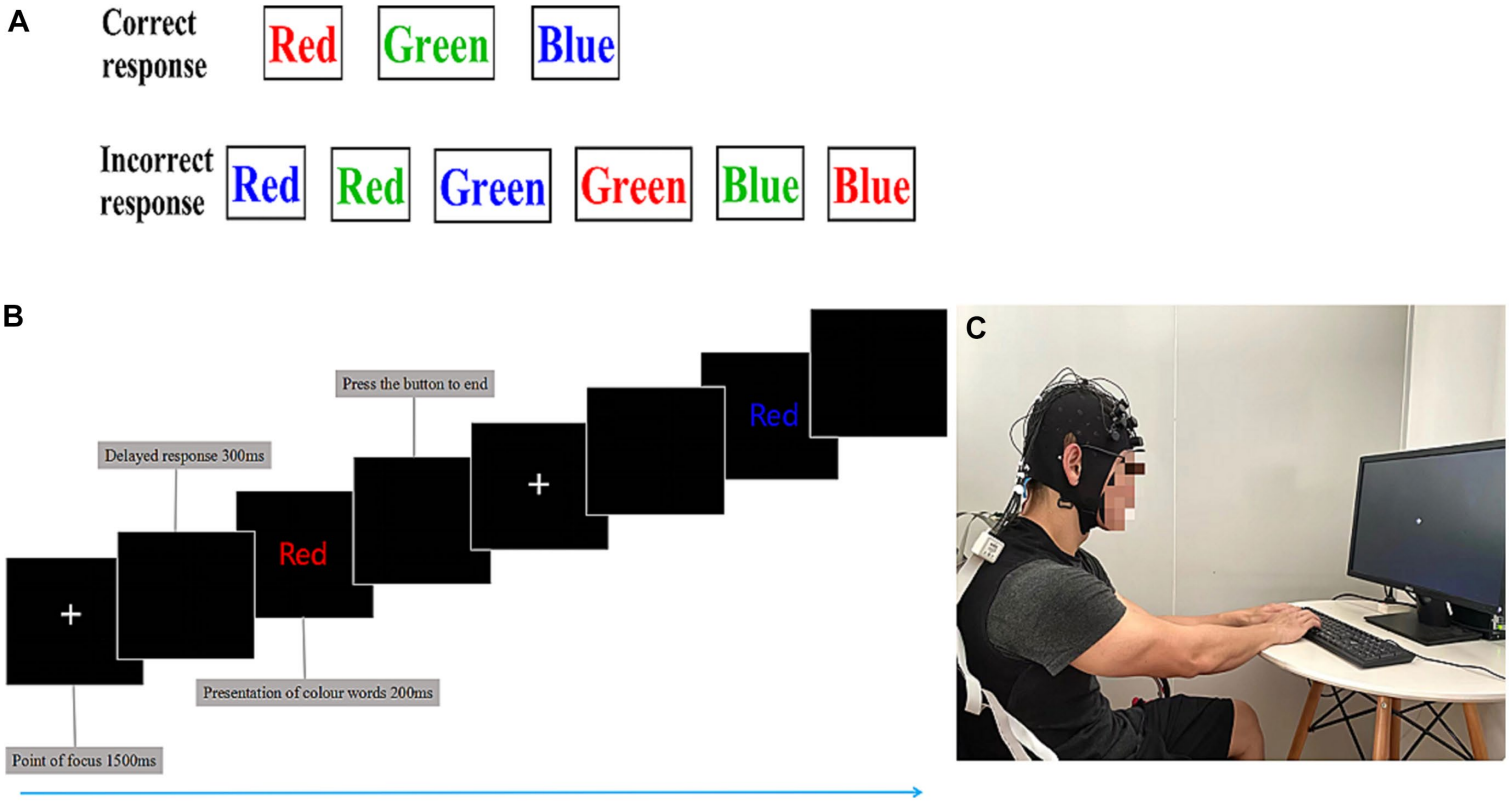
The color-word matching Stroop task. **(A)** Schematic representation of the color-word Stroop task (CWST). The task was divided into a correct response and an incorrect response. **(B)** Stroop task experiment flow. Using functional near-infrared spectroscopy (fNIRS), cortical hemodynamic changes were measured while athletes performed the CWST. Each presentation of colored words was 200 ms, and the delayed response was 300 ms, beginning with a 1,500 ms rest period, for a total of 24 trials, last 15 min. **(C)** For the Stroop tasks, the athletes were asked to stare at the central white plus sign (+); the prompt word for the CWST was in English, and the participants were asked to select the color that matched the meaning of the central word.

The Stroop task was designed with E-prime software, and the markers stimulating signal data of the brain were recorded with a NIRSport spectrometer (NIRSport, NIRxMedical Technology LLC, Glen Head, NY, United States). To avoid the interference of skin moisture-induced blood flow changes, as shown in Figure 2, athletes performed the CWST for 15 min before and after the swimming session. The obtained RT and ACC data were processed by E-data software (Kujach et al., 2018).

**Figure 3:**
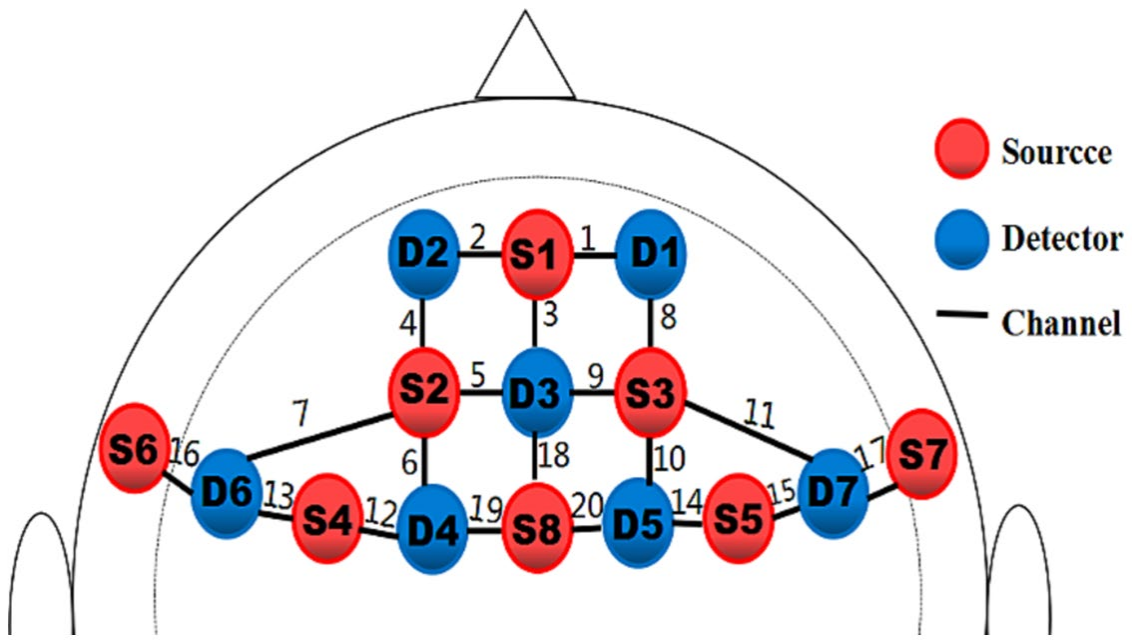
Schematic of the probe setup when positioned on the athlete’s head. The fNIRS system consisted of eight light sources (red) and seven detectors (blue) with a 3 cm source-detector separation, comprising 20 channels (black) across the prefrontal lobe.

With a montage design based on the fNIRS Optodes’ Location Decider (fOLD) and the brain ROIs distribution based on a high-density 10 × 10 international system, a portable near-infrared spectroscopy test imager (NIRSport, NIRxMedical Technology LLC, Glen Head, NY, United States) was placed on 8 brain regions of the athlete’s prefrontal lobe. The specific channel and brain region designs are shown in Table 2 and Figure 3 (Valer et al., 2007; Morais et al., 2018; Lacerenza et al., 2021). Data from fNIRS were recorded, the signal-to-noise ratio was assessed using NIRStar acquisition software, and the raw data were processed using nirsLAB according to the modified Beer–Lambert law (Slone et al., 2012; Ayache et al., 2014).

**Table 2:** The channel corresponds to the location of the prefrontal brain area.

Channel	Location	S-D	Brain location	ROIs
CH1	Fpz-Fp2	S1-D1	Ba10-Frontopolar area	R-FPA
CH2	Fpz-Fp1	S1-D2	Ba10-Frontopolar area	L-FPA
CH3	Fpz-AFz	S1-D3	Ba10-Frontopolar area	M-FPA
CH4	AF3-Fp1	S2-D2	Ba10-Frontopolar area	L-FPA
CH5	AF3-AFz	S2-D3	Ba10-Frontopolar area	L-FPA
CH6	AF3-F1	S2-D4	Ba9-Dorsolateral pefrontal cortex	L-DLFPC
CH7	AF3-F5	S2-D6	Ba46-Dorsolateral pefrontal cortex	L-DLFPC
CH8	AF4-Fp2	S3-D1	Ba10-Frontopolar area	R-FPA
CH9	AF4-AFz	S3-D3	Ba10-Frontopolar area	R-FPA
CH10	AF4-F2	S3-D5	Ba9-Dorsolateral pefrontal cortex	R-DLFPC
CH11	AF4-F6	S3-D7	Ba46-Dorsolateral pefrontal cortex	R-DLFPC
CH12	F3-F1	S4-D4	Ba9-Dorsolateral pefrontal cortex	L-DLFPC
CH13	F3-F5	S4-D6	Ba45-Pars triangularis Broca’s area	L-VLPFC
CH14	F4-F2	S5-D5	Ba9-Dorsolateral pefrontal cortex	R-DLFPC
CH15	F4-F6	S5-D7	Ba45-Pars triangularis Broca’s area	R-VLPFC
CH16	F7-F5	S6-D6	Ba45-Pars triangularis Broca’s area	L-VLPFC
CH17	F8-F6	S7-D7	Ba45-Pars triangularis Broca’s area	R-VLPFC
CH18	Fz-AFz	S8-D3	Ba9-Dorsolateral pefrontal cortex	M-DLFPC
CH19	Fz-F1	S8-D4	Ba9-Dorsolateral pefrontal cortex	L-DLFPC
CH20	Fz-F2	S8-D5	Ba9-Dorsolateral pefrontal cortex	R-DLFPC

R, right; L, left; M, middle; FPA, frontopolar area; DLFPC, dorsolateral prefrontal cortex; VLPFC, ventral prefrontal cortex.

### 2.6 Data analysis

All experimental data were statistically analyzed using SPSS 23.0 software, and the type I error was corrected using the alpha correction method. A paired sample t-test was used to analyze the related data. *p* < 0.05 indicated statistical significance. Data are expressed as the mean ± standard deviation, and GraphPad Prism 9.0 was used for graphical drawing.

## 3 Results

### 3.1 Athletic physical performance during the 1,500 m freestyle at maximal speed

#### 3.1.1 The segmentation performance

**Figure 4:**
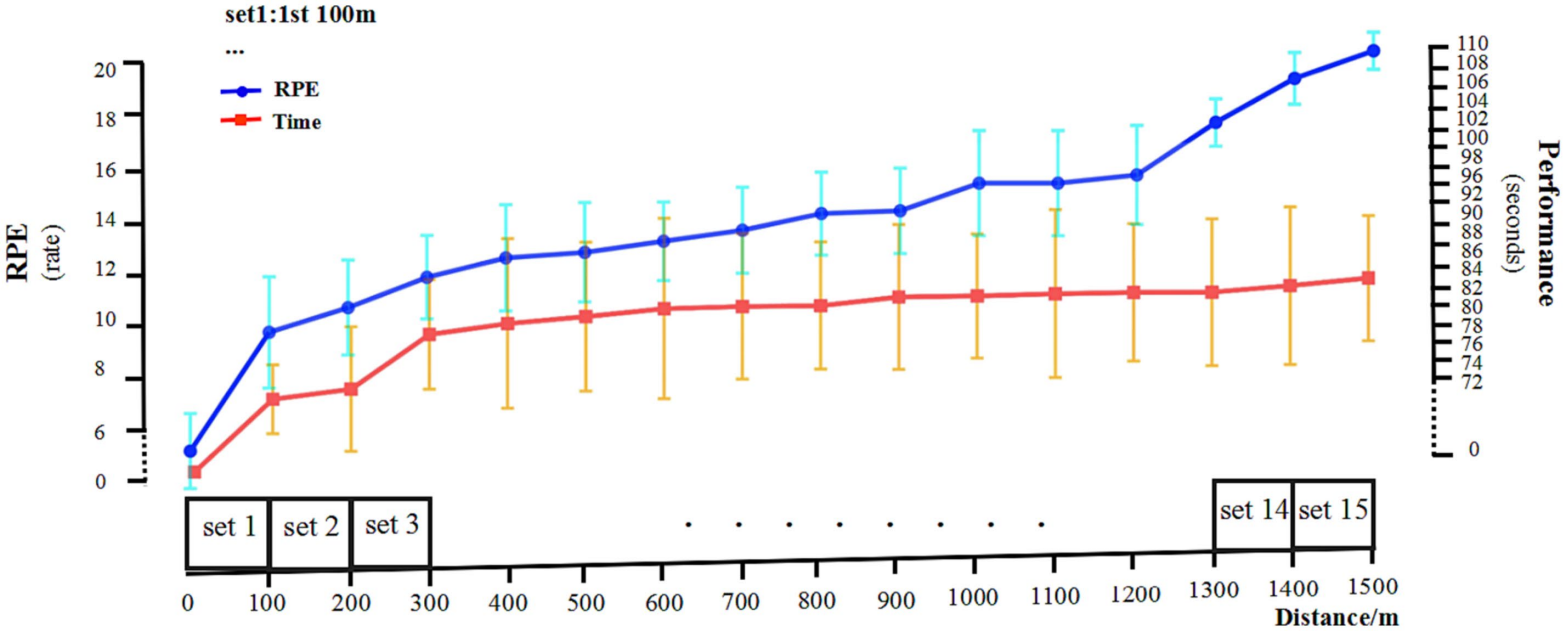
RPE and 100 m segmentation performance. RPE, Rating of Perceived Exertion. Performance, the time (seconds) of 100 m freestyle. Left *Y*-axis, RPE scores. Right *Y*-axis, segmentation performance for each 100 m.

As shown in Figure 4, during the freestyle, the athletes performed best in the first 100 m, followed by a downward trend. From the fifth 100 m, the performance was almost in a stable state with a relatively small range of fluctuations. In the last three 100 m of the 1,500 m freestyle at maximal speed, the athletes’ RPE value increased significantly (*p* < 0.01), reaching the level of 19–20. In summary, all athletes commenced the 1,500 m freestyle at maximal speed.

#### 3.1.2 The results of Bla, BUN, CK, 5-HT, and HR

**Figure 5:**
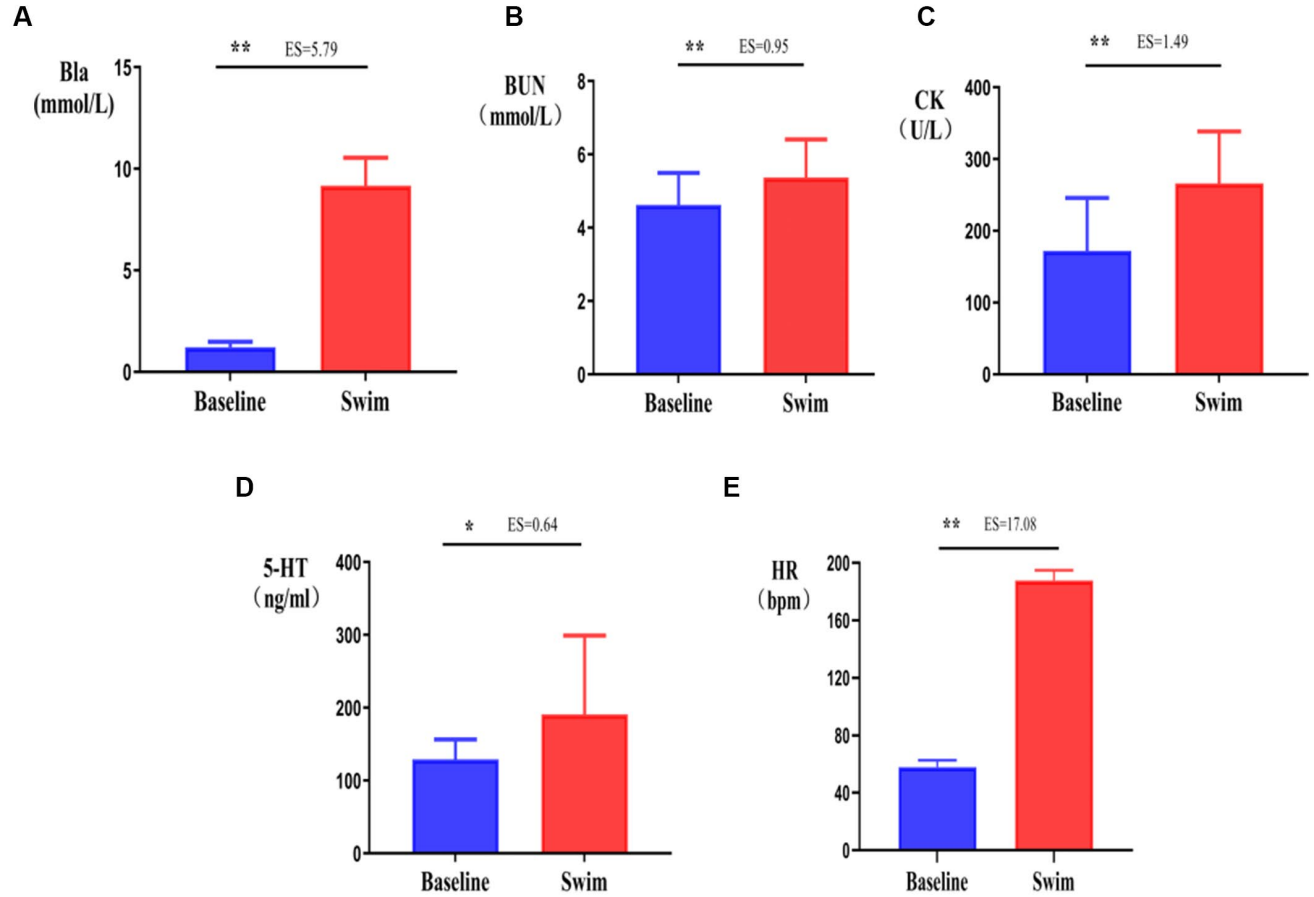
Changes in Bla, BUN, CK, 5-HT, and HR in athletes’ blood after the 1,500 m freestyle at maximal speed. ∗*p* < 0.05, ∗∗*p* < 0.01, compared with the baseline. **(A)** Bla: blood lactic acid, **(B)** CK: serum creatine kinase, **(C)** BUN: blood urea, **(D)** 5-HT: serotonin, **(E)** HR: Heart rate.

After the 1,500 m freestyle at maximal speed, Bla was significantly increased immediately (*p* < 0.01) (Figure 5A), reaching 11.2 mmol/L, and CK was significantly increased immediately (*p* < 0.05) (Figure 5B), reaching 340 U/L. Additionally, BUN was also increased significantly (*p* < 0.01) (Figure 5C). At the same time, the concentration of 5-HT, a neurotransmitter that reflects central nervous fatigue, increased significantly after exercise (*p* < 0.05) (Figure 5D), HR was increased significantly (*p* < 0.01) (Figure 5E), and reached approximately 200 bpm (HRmax). The results showed that the athletes were in a state of exercise-fatigue after the 1,500 m freestyle at maximal speed.

### 3.2 Cognitive function of athletes decreased after the 1,500 m freestyle at maximal speed

**Figure 6:**
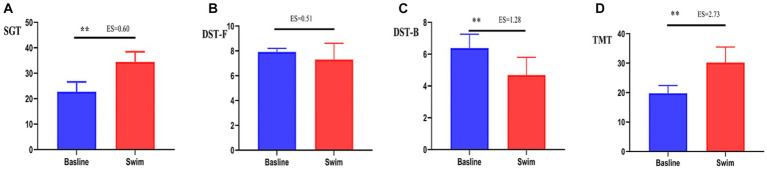
Changes in SGT, DST-F, DST-B, and TMT after the 1,500 m freestyle at maximal speed. ∗∗*p* < 0.01, compared with baseline. **(A)** SGT: Shuerte scale test, **(B)** DST-F: digit span test-forward, **(C)** DST-B: digit span test-backward, **(D)** TMT: Trail Making Test.

After the 1,500 m freestyle at maximal speed, the time consumed on the SGT and TMT significantly increased (*p* < 0.01) (Figures 6A,D), and the time spent performing the DST-B significantly decreased (*p* < 0.01) (Figure 6C), while the changes in the time spent performing the DST-F were not statistically significant (*p* > 0.05) (Figure 6B). This indicates that the cognitive function of athletes decreased after the 1,500 m freestyle at maximal speed.

### 3.3 Changes in the indicators of cognitive function measured by fNIRS after the 1,500 m freestyle at maximal speed

**Figure 7:**
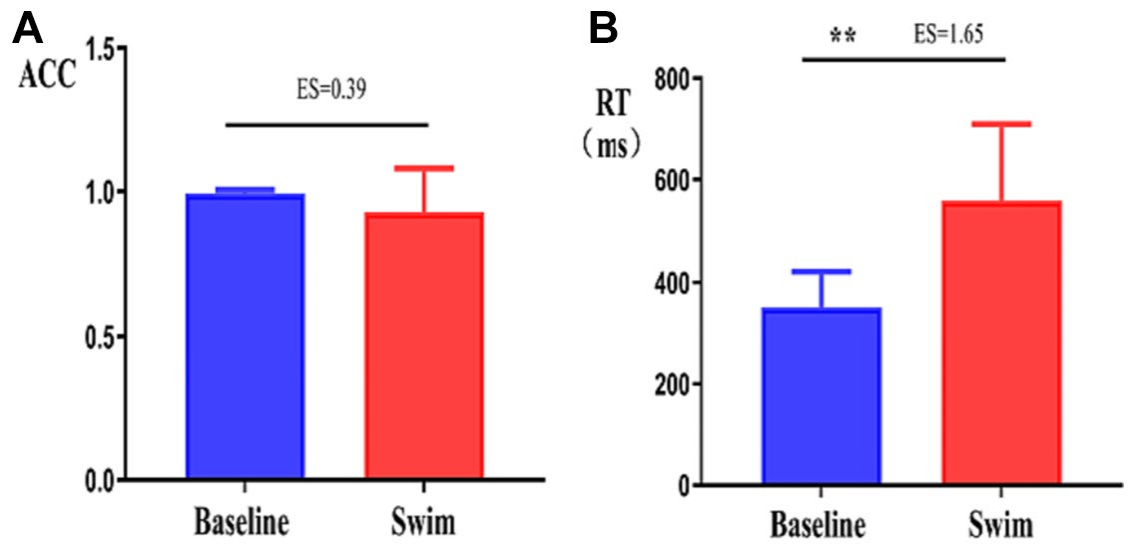
Changes in the accuracy and response time of the athletes during the Stroop task. ∗∗*p* < 0.01, comparison of accuracy rate (ACC, **A**), and response time (RT, **B**) between the correct response and incorrect response tasks.

ACC and RT are indicators of cognitive function. After the 1,500 m freestyle, the ACC of performing the CWST decreased slightly but with no statistical significance (*p* > 0.05) (Figure 7A). In contrast, the RT increased significantly (*p* < 0.01) (Figure 7B).

**Figure 8:**
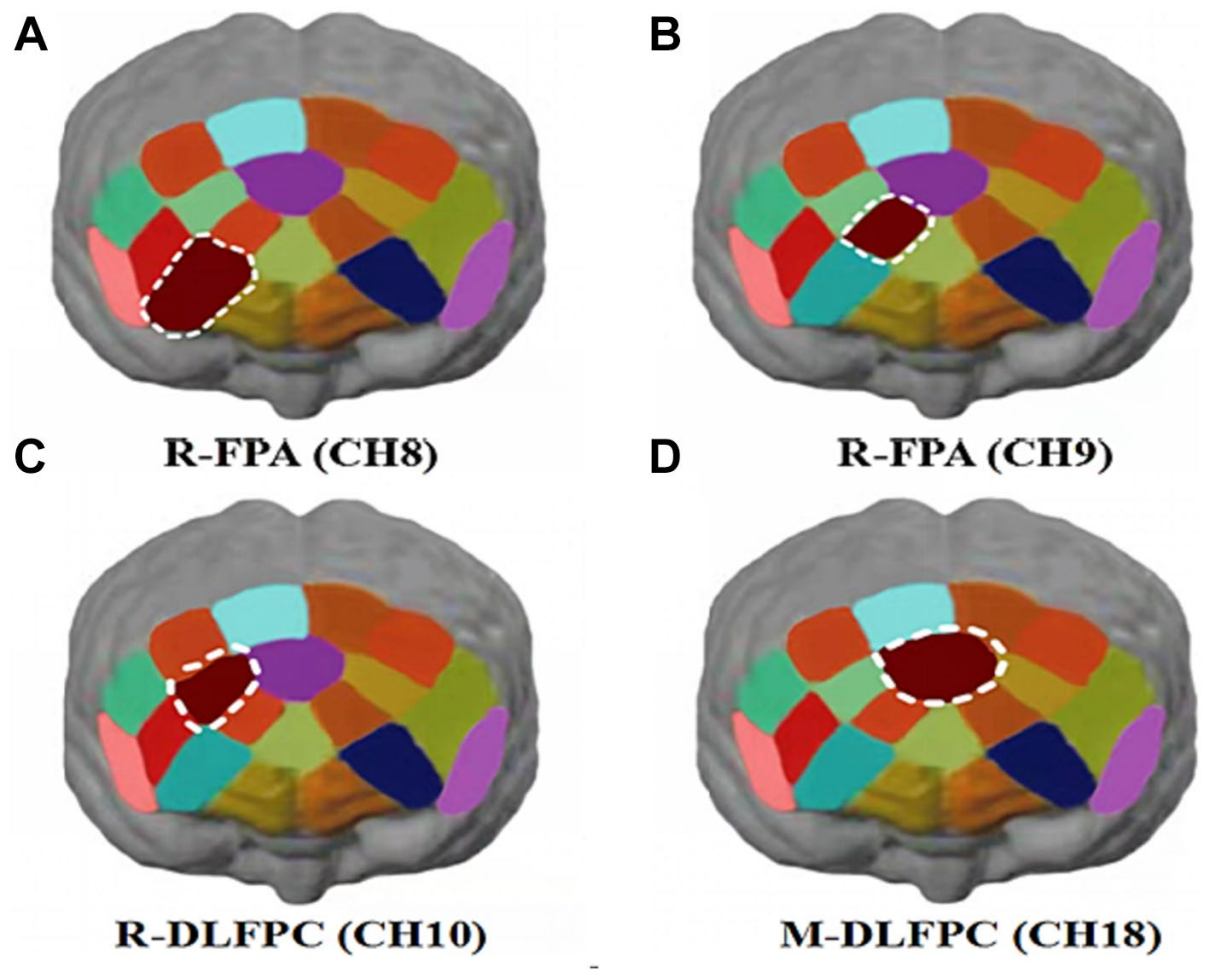
Changes in Oxy-Hb concentrations in prefrontal brain regions. Channels of ROIs activated in prefrontal cortical regions with greater response during the Stroop task after 1,500 m freestyle. Each of the small circles in different colors represents one channel, and the color scale represents the t-values at the group level. Each white circle represents an ROI. **(A)** Channel 8 of the right frontopolar area (R-FPA) (*p* < 0.01); **(B)** Channel 9 of R-FPA areas (*p* < 0.05); **(C)** Channel 10 of the right dorsolateral prefrontal cortex (R-DLPFC) (*p* < 0.05); **(D)** Channel 18 of the middle dorsolateral prefrontal cortex (M-DLPFC) (*p* < 0.01).

Hbdiff changes in athletes’ PFC after the 1,500 m freestyle at maximal speed. The Oxy-Hb concentration signal data of 20 channels in the PFC of athletes, CH8 (*p* < 0.01) and CH9 (*p* < 0.05) in the PFC of R-FPA (Figure 8A), and in the PFC of R-DLFPC (Figure 8B). The concentration of CH10 (*p* < 0.05) changed significantly (Figure 8C), and the t-value was significantly positively activated. The concentration of Oxy-Hb in CH18 (*p* < 0.01) located in the PFC of the M-DLFPC was significantly different (Figure 8D), and the t-value was negatively activated. The PFC of the athletes was activated after the 1,500 m freestyle at maximal speed, as shown in Table 3.

**Table 3:** Stroop-interference-related oxy-Hb changes in all ROIs of each channel.

Channel	S-D	ROIs	Baseline	Swim	*T*	*p*	ES
CH1	S1-D1	R-FPA	4.91 ± 9.95	0.43 ± 15.27	0.875	0.399	0.24
CH2	S1-D2	L-FPA	2.63 ± 9.83	−2.32 ± 17.36	0.888	0.392	0.25
CH3	S1-D3	M-FPA	3.37 ± 5.07	1.40 ± 14.16	0.501	0.625	0.14
CH4	S2-D2	L-FPA	2.46 ± 6.14	−2.16 ± 15.81	1.195	0.255	0.33
CH5	S2-D3	L-FPA	1.68 ± 3.55	−0.23 ± 5.19	1.410	0.184	0.39
CH6	S2-D4	L-DLFPC	1.46 ± 3.94	0.41 ± 3.60	1.324	0.210	0.37
CH7	S2-D6	L-DLFPC	3.34 ± 4.34	2.34 ± 5.75	0.590	0.566	0.16
CH8	S3-D1	R-FPA	5.33 ± 5.91	−8.34 ± 5.47	6.587	0.000^**^	1.83
CH9	S3-D3	R-FPA	3.74 ± 5.70	−2.80 ± 10.49	2.399	0.034^*^	0.67
CH10	S3-D5	R-DLFPC	2.60 ± 3.94	−0.71 ± 4.21	2.7	0.019^*^	0.75
CH11	S3-D7	R-DLFPC	2.59 ± 4.90	0.77 ± 6.80	1.092	0.296	0.30
CH12	S4-D4	L-DLFPC	2.95 ± 3.80	3.40 ± 7.73	−0.199	0.846	0.06
CH13	S4-D6	L-VLPFC	3.08 ± 6.90	0.50 ± 8.37	0.889	0.391	0.25
CH14	S5-D5	R-DLFPC	0.51 ± 4.45	−2.27 ± 6.28	1.187	0.258	0.33
CH15	S5-D7	R-VLPFC	2.50 ± 6.62	−0.44 ± 6.07	1.106	0.290	0.31
CH16	S6-D6	L-VLPFC	6.19 ± 9.98	3.33 ± 10.18	0.834	0.421	0.23
CH17	S7-D7	R-VLPFC	5.66 ± 10.03	1.72 ± 10.31	1.209	0.250	0.34
CH18	S8-D3	M-DLFPC	−0.62 ± 1.04	0.96 ± 0.74	−4.291	0.001^**^	1.19
CH19	S8-D4	L-DLFPC	2.18 ± 6.20	−1.75 ± 7.30	1.637	0.128	0.45
CH20	S8-D5	R-DLFPC	3.29 ± 6.24	1.32 ± 7.79	0.982	0.345	0.27

*p < 0.05.

**p < 0.01, compared with the baseline.

R, right; L, left; M, middle; FPA, frontopolar area; DLFPC: dorsolateral prefrontal cortex; VLPFC, ventral prefrontal cortex.

**Figure 9:**
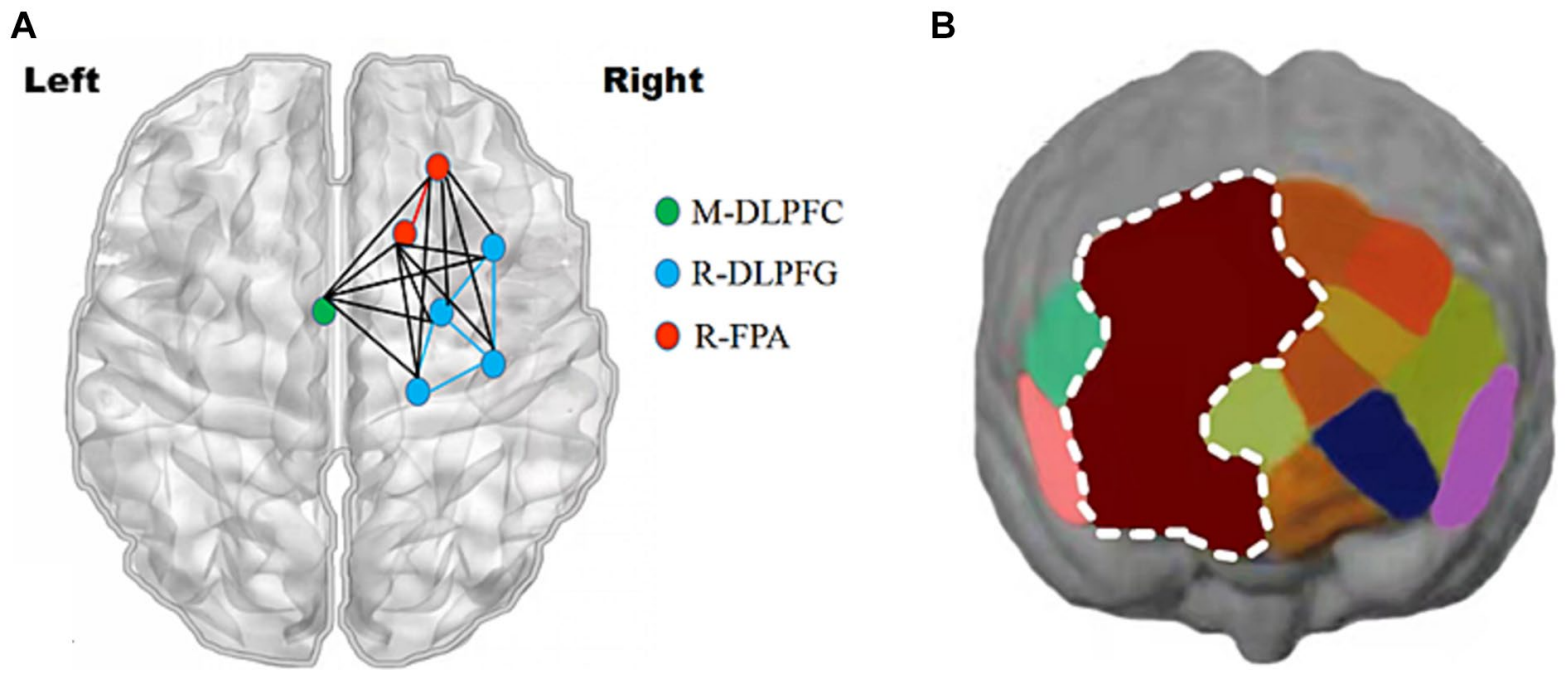
Changes in prefrontal brain regions. Activation patterns of the mirror neuron system (MNS) after 1,500 m freestyle when using the ROI-based group analysis. **(A)** Functional connectivity during the exercise state. Nodes with different colors refer to channels of ROIs, and the line between two nodes indicates an edge between these two channels. Note that one green node represents the middle dorsolateral prefrontal cortex (M-DLPFC), four blue nodes represent the right dorsolateral prefrontal cortex (R-DLPFC), and two red nodes represent the right frontopolar area (R-FPA). **(B)** ROIs of prefrontal cortical activation in response to Stroop tasks after 1,500 m freestyle. Small circles in the different colors represent channels, and the color scale represents the *t*-values at the group level. The white circle represents an ROI.

**Figure 10:**
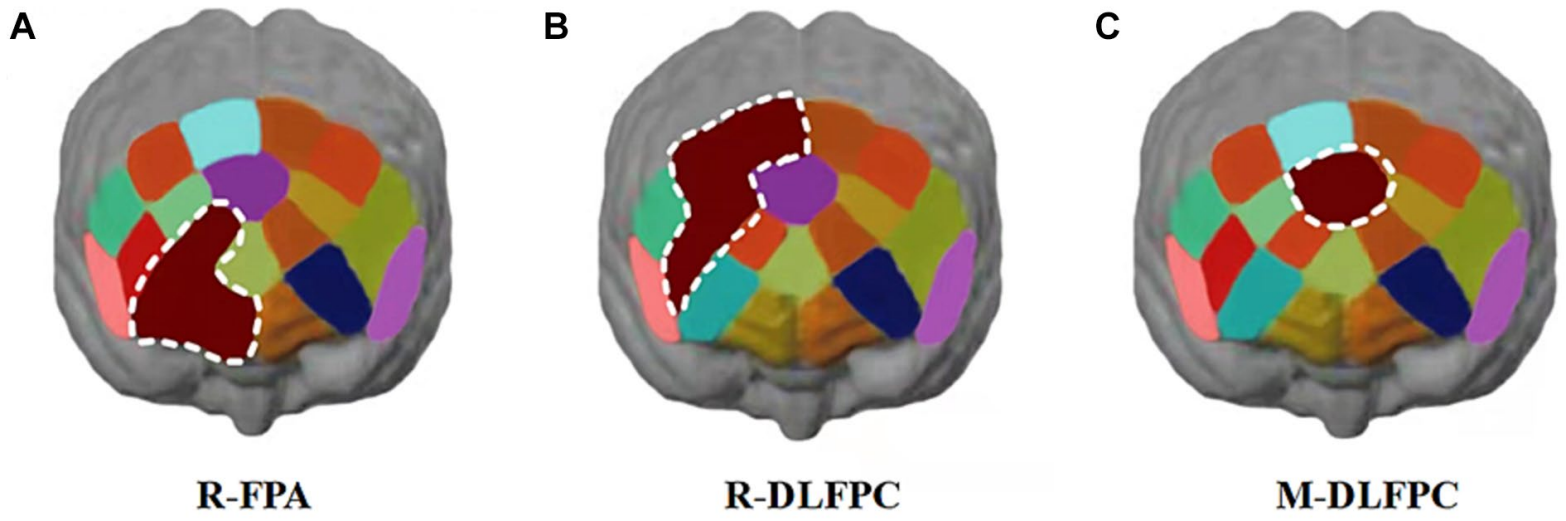
Cortical activation patterns during the color-word matching Stroop task. ROIs activated in the prefrontal cortex with a greater response during the Stroop task after 1,500 m freestyle (*p* < 0.01). Each small circle in the different colors represents one channel, and the color scale represents the *t*-values at the group level. Each white circle represents an ROI. **(A)** the right frontopolar area (R-FPA) of ROIs; **(B)** the right dorsolateral prefrontal cortex (R-DLPFC) of ROIs; **(C)** the middle dorsolateral prefrontal cortex (M-DLPFC) of ROIs.

Analysis of Oxy-Hb change concentration signal data from 20 channels in the PFC of athletes by brain ROIs (Figure 9B) showed that the total Oxy-Hb concentrations in the three ROIs, R-FPA (Figure 10A), R-DLFPC (Figure 10B), and M-DLFPC (Figure 10C), changed significantly (*p* < 0.01) (Figure 10B), as shown in Table 4.

**Table 4:** Changes in athletes’ 0xy-Hb indicators of ROIs.

Channel	ROIs	Baseline	Swim	*T*	*p*	ES
CH1-8-9	R-FPA	4.66 ± 7.28	−3.57 ± 11.50	3.920	0.000^**^	0.63
CH2-4-5	L-FPA	2.25 ± 6.82	−1.57 ± 13.55	1.696	0.098	0.27
CH3	M-FPA	3.37 ± 5.07	1.40 ± 14.16	0.501	0.625	0.14
CH6-7-12-19	L-DLFPC	2.48 ± 4.59	1.10 ± 6.43	1.461	0.150	0.20
CH10-11-14-20	R-DLFPC	2.24 ± 4.92	−0.22 ± 6.37	2.730	0.009^**^	0.38
CH13-16	L-VLPFC	4.63 ± 8.55	1.91 ± 9.25	1.236	0.228	0.24
CH15-17	R-VLPFC	4.08 ± 8.48	0.64 ± 8.36	1.668	0.108	0.33
CH18	M-DLFPC	−0.62 ± 1.04	0.96 ± 0.74	−4.291	0.001^**^	1.19

^**^p < 0.01, compared with Baseline. R, right; L, left; M, middle; FPA, frontopolar area; DLFPC, dorsolateral prefrontal cortex; VLPFC, ventral prefrontal cortex.

## 4 Discussion

In this study, we investigated the effects of a single 1,500 m freestyle at maximal speed on cognitive function and explored the changes in brain ROIs during freestyle at maximal speed-induced cognitive decline. The participants’ HR reached approximately 200 bpm (HRmax), RPE increased significantly to 20, Bla increased significantly after exercise, and performance decreased. Through the detection of inhibitory control ability in the Stroop task measured by fNIRS, the SGT, TMT, DST, and serum 5-HT, we found that 1,500 m freestyle at maximal speed enhanced the activation of three brain regions, R-FPA, R-DLFPC, and M-DLFPC, in brain ROIs, increasing Stroop interference RT. Athletes who performed DST-B, TMT, and SGT tests were significantly less efficient, and serum 5-HT was significantly increased. Our data suggest that the network of brain regions in the right prefrontal cortex was activated and cognitive function decreased after the 1,500 m freestyle at maximal speed. This study helps to uncover the potential changes by which freestyle with high intensity reduces cognitive function in athletes. Several points were discussed here, including intensity- and workload-related dose–response effects on cognitive function and mechanisms of cognition decline measured by fNIRS.

Inhibitory control (IC) during the Stroop task can be detected to assess the cognitive function of the PFC (Yang et al., 2020; Qi et al., 2021). Moderate-intensity aerobic exercise for a period of 20 min immediately improved inhibitory control significantly (Kraemer et al., 2002; Chang et al., 2015). Moderate-intensity acute resistance exercise for 30 min not only increased RT and ACC behavioral performance of Go/No-Go tasks but also selectively enhanced executive cognitive functions of IC and attention. Furthermore, 60 min of acute moderate-intensity exercise had a great effect on IC and immediately improved cognitive function in the Stroop task but did not affect working memory or cognitive flexibility in young adults (Chang and Etnier, 2009a; Tsukamoto et al., 2017). These studies showed that the cognitive performance of IC and other executive functions (EF) were improved after moderate-intensity aerobic exercise (Lambourne and Tomporowski, 2010; Ludyga et al., 2016).

However, it is notable that the domain-specific cognitive function and affective response are impacted by intensity- and workload-related dose–response effects of acute resistance exercise (Chang and Etnier, 2009b; Engeroff et al., 2019). High-intensity exercise increased the RT of executive tasks and decreased behavioral performance as well as cognitive performance (Drollette et al., 2012; Tsai et al., 2014). Moderate- and high-intensity acute resistance exercise for a period of 40 min immediately improved cognitive performance on the Stroop task and significantly improved IC, while low-intensity exercise had little effect (Tsukamoto et al., 2017). Furthermore, 45 min of acute high-intensity resistance exercise reduced behavioral performance on the Stroop task but increased RT, whereas 180 min of moderate-intensity exercise improved behavioral performance on the Simon task and cognitive EF (Brush et al., 2016). Most reports have shown that acute moderate-intensity resistance exercise can cause cognitive function decline (Hirano et al., 2013; Russell et al., 2021; Engeroff et al., 2022). Consistent with the literature, this research found that the 1,500 m freestyle at maximal speed led to a decrease in cognitive function; IC in the Stroop task, SGT, and TMT were reduced, and serum 5-HT increased, which is possibly due to central fatigue and cognitive decline, affecting the effective contraction of skeletal muscle (Kieran et al., 2018; Zhai et al., 2018).

Previous studies have reported the mechanisms of cognition decline in extreme exercise (Ando et al., 2022). PFC activation during acute exercise measured by fNIRS enhanced cerebral blood flow to the DLPFC and FPA and improved performance in Stroop tasks requiring IC capabilities (Fujihara et al., 2021). Improving the FC activity of ROIs can better regulate the decline in sports ability caused by the decline in IC capabilities (Radel et al., 2017). Our research results are consistent with those of previous reports. It is worth noting that in this study, three ROIs of the FPA, DLPFC, and M-DLPFC on the right side of the brain were activated, and the functional connectivity (FC) of the PFC spatial grid was altered after the 1,500 m freestyle at maximal speed. Acute exercise affects cognitive function by altering neural processing and changes in network patterns between multiple brain regions (Moriarty et al., 2019; Urquhart et al., 2019; Burin et al., 2020). Whether the change in brain cognitive function caused by freestyle at maximal speed is related to sports fatigue depends on the activation of the DLPFC (Ludyga et al., 2019). Understanding the mechanism of the decline in sports function caused by high-intensity exercise could provide a basis for improving sports economy and preventing cognitive function damage (Rhee and Mehta, 2018).

The DLPFC is closely related to the sensorimotor cortex and is responsible for cognitive control and goal-oriented behavior (Causse et al., 2018). A high intensity of cycling until exhaustion was found to increase DLPFC activity in healthy adults (Bediz et al., 2016). When cycling to fatigue at 60% maximum aerobic exercise capacity (PAP) intensity, the oxy-hemoglobin (Δ[HbO]) concentration of R-DLPFC decreased (Fox et al., 2009). Changes in the oxy-hemoglobin (Δ[HbO]) of the DLPFC not only delay the occurrence of fatigue but also improve cognitive function (Ji et al., 2019). Although both the right and left PFC were activated by the planning task, activation of the right DLPFC was more sensitive to executive task difficulty (Newman et al., 2003). The activation of the DLPFC may reflect interference processing and inhibition of response, and the left DLPFC may be the neural matrix to improve Stroop performance (Yanagisawa et al., 2010). This finding suggests that the changes in Δ[HbO] in the R-DLPFC and R-FPA during the Stroop task activated the right PFC brain region.

In summary, the 1,500 m freestyle at maximal speed led to cognitive function decline and fatigue. The potential mechanism might be the reduction in the cognitive function of athletes due to the activation of the R-FPA, R-DLPFC, and M-DLPFC regions of interest in the PFC and functional connectivity. This study is helpful for understanding the relationship between the decline in cognitive function and exercise fatigue.

## 5 Conclusion

A single 1,500 m freestyle at maximal speed led to fatigue and cognitive function decline. The potential mechanism might be the activation of the R-FPA, R-DLPFC, and M-DLPFC regions of interest in the PFC and functional connectivity.

## 6 Limitations and future research

Several limitations of this study warrant consideration. Firstly, the inclusion of solely male athletes restricts the generalizability of the findings. Secondly, the utilization of a probe in fNIRS measurements that solely encompassed the prefrontal lobe resulted in the inability to detect deeper subcortical regions like the hippocampus. Thirdly, this study lacks correlation analysis of fatigue and cognitive function. Consequently, the consistency of the enduring effects of exercise on cognitive function across various age groups remains uncertain ^[80]^. Consequently, future studies should investigate cognitive function in response to diverse intensities of fatiguing exercise and among different individuals.

## Data availability statement

The datasets presented in this study can be found in online repositories. The names of the repository/repositories and accession number (s) can be found in the article/supplementary material.

## Ethics statement

The studies involving humans were approved by Ethics Committee of Guangzhou Sport University. The studies were conducted in accordance with the local legislation and institutional requirements. The participants provided their written informed consent to participate in this study. Written informed consent was obtained from the individual (s) for the publication of any potentially identifiable images or data included in this article.

## Author contributions

ZL: Writing – original draft. WH: Investigation, Writing – review & editing. WL: Supervision, Writing – review & editing. XW: Supervision, Validation, Writing – review & editing. YM: Visualization, Writing – review & editing. GX: Funding acquisition, Methodology, Writing – original draft, Writing – review & editing.

## References

[ref1] Amjad I. Toor H. Niazi I. K. Afzal H. Jochumsen M. Shafique M.. (2019). Therapeutic effects of aerobic exercise on EEG parameters and higher cognitive functions in mild cognitive impairment patients[J]. Int. J. Neurosci. 129, 551–562. doi: 10.1080/00207454.2018.1551894, PMID: 30929591

[ref2] Ando S. Komiyama T. Tanoue Y. Sudo M. Costello J. T. Uehara Y.. (2022). Cognitive improvement after aerobic and resistance exercise is not associated with peripheral biomarkers[J]. Front. Behav. Neurosci. 16:150. doi: 10.3389/fnbeh.2022.853150, PMID: 35368295 PMC8967356

[ref3] Ayache S. S. Al-Ani T. Lefaucheur J.-P. (2014). Distinction between essential and physiological tremor using Hilbert-Huang transform[J]. Neurophysiol. Clin. 44, 203–212. doi: 10.1016/j.neucli.2014.03.006, PMID: 24930942

[ref4] Bediz C. S. Oniz A. Guducu C. Ural Demirci E. Ogut H. Gunay E.. (2016). Acute supramaximal exercise increases the brain oxygenation in relation to cognitive workload[J]. Front. Hum. Neurosci. 10:771. doi: 10.3389/fnhum.2016.00174, PMID: 27148022 PMC4837702

[ref5] Blumenthal J. A. Smith P. J. Mabe S. Hinderliter A. Welsh-Bohmer K. Browndyke J. N.. (2020). Longer-term effects of diet and exercise on Neurocognition: 1-year follow-up of the enlighten trial[J]. J. Am. Geriatr. Soc. 68, 559–568. doi: 10.1111/jgs.16252, PMID: 31755550 PMC7056586

[ref6] Borg G. (1982). Psychophysical basis of perceived exertion[J]. Med. Sci. Sports Exerc. 14, 477–480. doi: 10.1249/00005768-198205000-00012, PMID: 7154893

[ref7] Brisswalter J. Arcelin R. Audiffren M. Delignières D. (1997). Influence of physical exercise on simple reaction time: effect of physical fitness[J]. Percept. Mot. Skills 85, 1019–1027. doi: 10.2466/pms.1997.85.3.1019, PMID: 9399313

[ref8] Broadhouse K. M. Singh M. F. Suo C. Gates N. Wen W. Brodaty H.. (2020). Hippocampal plasticity underpins long-term cognitive gains from resistance exercise in MCI[J]. NeuroImage 25:102182. doi: 10.1016/j.nicl.2020.102182 31978826 PMC6974789

[ref9] Brush C. J. Olson R. L. Ehmann P. J. Osovsky S. Alderman B. L. (2016). Dose–response and time course effects of acute resistance exercise on executive function[J]. J. Sport Exerc. Psychol. 38, 396–408. doi: 10.1123/jsep.2016-0027, PMID: 27385719

[ref10] Burin D. Liu Y. Yamaya N. Kawashima R. (2020). Virtual training leads to physical, cognitive and neural benefits in healthy adults[J]. NeuroImage 222:117297. doi: 10.1016/j.neuroimage.2020.117297, PMID: 32828927

[ref11] Causse M. Chua Z. Peysakhovich V. del Campo N. Matton N. (2018). Author correction: mental workload and neural efficiency quantified in the prefrontal cortex using fNIRS[J]. Sci. Rep. 8:7184. doi: 10.1038/s41598-018-25614-2, PMID: 29717193 PMC5931575

[ref12] Chang Y. K. Chu C. H. Wang C. C. Wang Y. C. Song T. F. Tsai C. L.. (2015). Dose-response relation between exercise duration and cognition[J]. Med. Sci. Sports Exerc. 47, 159–165. doi: 10.1249/MSS.0000000000000383, PMID: 24870572

[ref13] Chang Y. K. Etnier J. L. (2009a). Effects of an acute bout of localized resistance exercise on cognitive performance in middle-aged adults: a randomized controlled trial study[J]. Psychol. Sport Exerc. 10, 19–24. doi: 10.1016/j.psychsport.2008.05.004

[ref14] Chang Y. K. Etnier J. L. (2009b). Exploring the dose-response relationship between resistance exercise intensity and cognitive function[J]. J. Sport Exerc. Psychol. 31, 640–656. doi: 10.1123/jsep.31.5.640, PMID: 20016113

[ref15] Chang Y. K. Tsai C. L. Huang C. C. Wang C. C. Chu I. H. (2014). Effects of acute resistance exercise on cognition in late middle-aged adults: general or specific cognitive improvement?[J]. J. Sci. Med. Sport 17, 51–55. doi: 10.1016/j.jsams.2013.02.007 23491140

[ref16] Chen T. Yue G. H. Tian Y. Jiang C. (2016). Baduanjin mind-body intervention improves the executive control function[J]. Front. Psychol. 7:7. doi: 10.3389/fpsyg.2016.02015, PMID: 28133453 PMC5233682

[ref17] Coetsee C. Terblanche E. (2017). The effect of three different exercise training modalities on cognitive and physical function in a healthy older population[J]. Eur. Rev. Aging Phys. Act. 14, 13–10. doi: 10.1186/s11556-017-0183-5, PMID: 28811842 PMC5553712

[ref18] Cooper S. B. Dring K. J. Morris J. G. Sunderland C. Bandelow S. Nevill M. E. (2018). High intensity intermittent games-based activity and adolescents’ cognition: moderating effect of physical fitness[J]. BMC Public Health 18, 1–14. doi: 10.1186/s12889-018-5514-6, PMID: 29739386 PMC5941716

[ref19] de la Rosa A. Olaso-Gonzalez G. Arc-Chagnaud C. Millan F. Salvador-Pascual A. García-Lucerga C.. (2020). Physical exercise in the prevention and treatment of Alzheimer's disease[J]. J. Sport Health Sci. 9, 394–404. doi: 10.1016/j.jshs.2020.01.004, PMID: 32780691 PMC7498620

[ref20] Devenney K. E. Guinan E. M. Kelly I. M. Mota B. C. Walsh C. Olde Rikkert M.. (2019). Acute high-intensity aerobic exercise affects brain-derived neurotrophic factor in mild cognitive impairment: a randomised controlled study[J]. BMJ Open Sport Exerc. Med. 5:e000499. doi: 10.1136/bmjsem-2018-000499, PMID: 31258928 PMC6563898

[ref21] Drollette E. S. Shishido T. Pontifex M. B. Hillman C.. (2012). Maintenance of cognitive control during and after walking in preadolescent children[J]. Med. Sci. Sports Exerc. 44, 2017–2024. doi: 10.1249/MSS.0b013e318258bcd5, PMID: 22525770

[ref22] Engeroff T. Banzer W. Niederer D. (2022). The impact of regular activity and exercise intensity on the acute effects of resistance exercise on cognitive function[J]. Scand. J. Med. Sci. Sports 32, 94–105. doi: 10.1111/sms.14050, PMID: 34533869

[ref23] Engeroff T. Niederer D. Vogt L. Banzer W. (2019). Intensity and workload related dose-response effects of acute resistance exercise on domain-specific cognitive function and affective response–a four-armed randomized controlled crossover trial[J]. Psychol. Sport Exerc. 43, 55–63. doi: 10.1016/j.psychsport.2018.12.009

[ref24] Fox M. D. Zhang D. Snyder A. Z. Raichle M. E. (2009). The global signal and observed anticorrelated resting state brain networks[J]. J. Neurophysiol. 101, 3270–3283. doi: 10.1152/jn.90777.2008, PMID: 19339462 PMC2694109

[ref25] Fujihara H. Megumi A. Yasumura A. (2021). The acute effect of moderate-intensity exercise on inhibitory control and activation of prefrontal cortex in younger and older adults[J]. Exp. Brain Res. 239, 1765–1778. doi: 10.1007/s00221-021-06086-9, PMID: 33783561

[ref26] Herold F. Törpel A. Schega L. Müller N. G. (2019). Functional and/or structural brain changes in response to resistance exercises and resistance training lead to cognitive improvements–a systematic review[J]. Eur. Rev. Aging Phys. Act. 16, 1–33. doi: 10.1186/s11556-019-0217-2 31333805 PMC6617693

[ref27] Hirano Y. Obata T. Takahashi H. Tachibana A. Kuroiwa D. Takahashi T.. (2013). Effects of chewing on cognitive processing speed[J]. Brain Cogn. 81, 376–381. doi: 10.1016/j.bandc.2012.12.002, PMID: 23375117

[ref28] Hortobágyi T. Granacher U. Fernandez-del-Olmo M. Howatson G. Manca A. Deriu F.. (2021). Functional relevance of resistance training-induced neuroplasticity in health and disease[J]. Neurosci. Biobehav. Rev. 122, 79–91. doi: 10.1016/j.neubiorev.2020.12.019, PMID: 33383071

[ref29] Ji Z. Feng T. Mei L. Li A. Zhang C. (2019). Influence of acute combined physical and cognitive exercise on cognitive function: an NIRS study[J]. PeerJ 7:e7418. doi: 10.7717/peerj.7418, PMID: 31396453 PMC6681798

[ref30] Jin X. Wang L. Liu S. Zhu L. Loprinzi P. D. Fan X. (2019). The impact of mind-body exercises on motor function, depressive symptoms, and quality of life in Parkinson’s disease: a systematic review and meta-analysis[J]. Int. J. Environ. Res. Public Health 17:31. doi: 10.3390/ijerph17010031, PMID: 31861456 PMC6981975

[ref31] Kao S. C. Drollette E. S. Ritondale J. P. Khan N. Hillman C. H. (2018). The acute effects of high-intensity interval training and moderate-intensity continuous exercise on declarative memory and inhibitory control[J]. Psychol. Sport Exerc. 38, 90–99. doi: 10.1016/j.psychsport.2018.05.011

[ref32] Kao S. C. Westfall D. R. Soneson J. Gurd B. Hillman C. H. (2017). Comparison of the acute effects of high-intensity interval training and continuous aerobic walking on inhibitory control[J]. Psychophysiology 54, 1335–1345. doi: 10.1111/psyp.12889, PMID: 28480961

[ref33] Khan S. A. Sathyanarayan A. Mashek M. T. Ong K. T. Wollaston-Hayden E. E. Mashek D. G. (2015). ATGL-catalyzed lipolysis regulates SIRT1 to control PGC-1alpha/PPAR-alpha signaling[J]. Diabetes 64, 418–426. doi: 10.2337/db14-0325, PMID: 25614670 PMC4303962

[ref34] Kieran O. Peter B. O. Tim J. G. (2018). Pain and fatigue in sport: are they so different?[J]. Br. J. Sports Med. 52, 10–1136. 10.1136/bjsports-2017-098159 29051168

[ref35] Kraemer W. J. Adams K. Cafarelli E. Dudley G. A. Dooly C. Feigenbaum M. S.. (2002). American College of Sports Medicine position stand. Progression models in resistance training for healthy adults[J]. Med. Sci. Sports Exerc. 34, 364–380. doi: 10.1097/00005768-200202000-00027, PMID: 11828249

[ref36] Kujach S. Byun K. Hyodo K. Suwabe K. Fukuie T. Laskowski R.. (2018). A transferable high-intensity intermittent exercise improves executive performance in association with dorsolateral prefrontal activation in young adults[J]. NeuroImage 169, 117–125. doi: 10.1016/j.neuroimage.2017.12.003, PMID: 29203453

[ref37] Lacerenza M. Spinelli L. Buttafava M. Dalla Mora A. Zappa F. Pifferi A.. (2021). Monitoring the motor cortex hemodynamic response function in freely moving walking subjects: a time-domain fNIRS pilot study[J]. Neurophotonics 8:015006. doi: 10.1117/1.NPh.8.1.015006, PMID: 33628861 PMC7899043

[ref38] Lambourne K. Tomporowski P. (2010). The effect of exercise-induced arousal on cognitive task performance: a meta-regression analysis[J]. Brain Res. 1341, 12–24. doi: 10.1016/j.brainres.2010.03.091, PMID: 20381468

[ref39] Li M. Fang Q. Li J. Zheng X. Tao J. Yan X.. (2015). The effect of Chinese traditional exercise-Baduanjin on physical and psychological well-being of college students: a randomized controlled trial[J]. PLoS One 10:e0130544. doi: 10.1371/journal.pone.0130544, PMID: 26158769 PMC4497728

[ref40] Liu-Ambrose T. Nagamatsu L. S. Voss M. W. Khan K. M. Handy T. C. (2012). Resistance training and functional plasticity of the aging brain: a 12-month randomized controlled trial[J]. Neurobiol. Aging 33, 1690–1698. doi: 10.1016/j.neurobiolaging.2011.05.010, PMID: 21741129

[ref41] Ludyga S. Gerber M. Brand S. Holsboer-Trachsler E. Pühse U. (2016). Acute effects of moderate aerobic exercise on specific aspects of executive function in different age and fitness groups: a meta-analysis[J]. Psychophysiology 53, 1611–1626. doi: 10.1111/psyp.12736, PMID: 27556572

[ref42] Ludyga S. Mücke M. Colledge F. Pühse U. Gerber M. (2019). A combined EEG-fNIRS study investigating mechanisms underlying the association between aerobic fitness and inhibitory control in young adults - ScienceDirect[J]. Neuroscience 419, 23–33. doi: 10.1016/j.neuroscience.2019.08.045, PMID: 31487542

[ref43] Matthews M. J. Green D. Matthews H. Swanwick E. (2017). The effects of swimming fatigue on shoulder strength, range of motion, joint control, and performance in swimmers[J]. Phys. Ther. Sport 23, 118–122. doi: 10.1016/j.ptsp.2016.08.011, PMID: 27769804

[ref44] McMorris T. (2021). The acute exercise-cognition interaction: from the catecholamines hypothesis to an interoception model[J]. Int. J. Psychophysiol. 170, 75–88. doi: 10.1016/j.ijpsycho.2021.10.005, PMID: 34666105

[ref45] McMorris T. Hale B. J. (2012). Differential effects of differing intensities of acute exercise on speed and accuracy of cognition: a meta-analytical investigation[J]. Brain Cogn. 80, 338–351. doi: 10.1016/j.bandc.2012.09.001, PMID: 23064033

[ref46] Morais G. Z. Balardin J. B. Sato J. R. (2018). FNIRS Optodes' location decider (fOLD): a toolbox for probe arrangement guided by brain regions-of-interest[J]. Sci. Rep. 8:3341. doi: 10.1038/s41598-018-21716-z, PMID: 29463928 PMC5820343

[ref47] Moriarty T. Bourbeau K. Bellovary B. Zuhl M. N. (2019). Exercise intensity influences prefrontal cortex oxygenation during cognitive testing[J]. Behav. Sci. 9, 82–83. doi: 10.3390/bs9080083, PMID: 31357450 PMC6721405

[ref48] Newman S. D. Carpenter P. A. Varma S. Just M. A. (2003). Frontal and parietal participation in problem solving in the tower of London: fMRI and computational modeling of planning and high-level perception[J]. Neuropsychologia 41, 1668–1682. doi: 10.1016/S0028-3932(03)00091-5, PMID: 12887991

[ref49] Northey J. M. Cherbuin N. Pumpa K. L. Smee D. J. Rattray B. (2018). Exercise interventions for cognitive function in adults older than 50: a systematic review with meta-analysis[J]. Br. J. Sports Med. 52, 154–160. doi: 10.1136/bjsports-2016-096587, PMID: 28438770

[ref50] Pérez-Sousa M. Á. del Pozo-Cruz J. Olivares P. R. Cano-Gutiérrez C. A. Izquierdo M. Ramírez-Vélez R. (2021). Role for physical fitness in the association between age and cognitive function in older adults: a mediation analysis of the SABE Colombia study[J]. Int. J. Environ. Res. Public Health 18:751. doi: 10.3390/ijerph18020751, PMID: 33477293 PMC7829928

[ref51] Qi L. Yin Y. Bu L. Tang Z. Tang L. Dong G. (2021). Acute VR competitive cycling exercise enhanced cortical activations and brain functional network efficiency in MA-dependent individuals[J]. Neurosci. Lett. 757:135969. doi: 10.1016/j.neulet.2021.135969, PMID: 34023411

[ref52] Radel R. Brisswalter J. Perrey S. (2017). Saving mental effort to maintain physical effort: a shift of activity within the prefrontal cortex in anticipation of prolonged exercise[J]. Cogn. Affect. Behav. Neurosci. 17, 305–314. doi: 10.3758/s13415-016-0480-x, PMID: 27858329

[ref53] Rehfeld K. Müller P. Aye N. Schmicker M. Dordevic M. Kaufmann J.. (2017). Dancing or fitness sport? The effects of two training programs on hippocampal plasticity and balance abilities in healthy seniors[J]. Front. Hum. Neurosci. 11:305. doi: 10.3389/fnhum.2017.00305, PMID: 28674488 PMC5475381

[ref54] Rhee J. Mehta R. K. (2018). Functional connectivity during handgrip motor fatigue in older adults is obesity and sex-specific.[J]. Front. Hum. Neurosci. 12:455. doi: 10.3389/fnhum.2018.00455, PMID: 30483085 PMC6243051

[ref55] Russell B. McDaid A. Toscano W. Hume P. (2021). Predicting fatigue in long Duration Mountain events with a single sensor and deep learning model[J]. Sensors 21:442. doi: 10.3390/s21165442 34450884 PMC8399921

[ref56] Simard D. M. Reekum R. V. (1999). Memory assessment in studies of cognition-enhancing drugs for Alzheimer's disease.[J]. Drugs Aging 14, 197–230. doi: 10.2165/00002512-199914030-00004, PMID: 10220105

[ref57] Slone E. Westwood E. Dhaliwal H. Federico P. Dunn J. F. (2012). Near-infrared spectroscopy shows preictal hemodynamic changes in temporal lobe epilepsy[J]. Epileptic Disord. 14, 371–378. doi: 10.1684/epd.2012.0535, PMID: 23247924

[ref58] Song D. Doris S. F. (2019). Effects of a moderate-intensity aerobic exercise program on the cognitive function and quality of life of community-dwelling elderly people with mild cognitive impairment: a randomized controlled trial[J]. Int. J. Nurs. Stud. 93, 97–105. doi: 10.1016/j.ijnurstu.2019.02.019, PMID: 30901716

[ref59] Soysal P. Hurst C. Demurtas J. Firth J. Howden R. Yang L.. (2021). Handgrip strength and health outcomes: umbrella review of systematic reviews with meta-analyses of observational studies[J]. J. Sport Health Sci. 10, 290–295. doi: 10.1016/j.jshs.2020.06.009, PMID: 32565244 PMC8167328

[ref60] Tao J. Chen X. Liu J. Egorova N. Xue X. Liu W.. (2017). Tai chi Chuan and Baduanjin mind-body training changes resting-state low-frequency fluctuations in the frontal lobe of older adults: a resting-state fMRI study[J]. Front. Hum. Neurosci. 11:514. doi: 10.3389/fnhum.2017.00514, PMID: 29163096 PMC5670503

[ref61] Tomoto T. Tarumi T. Chen J. N. Hynan L. S. Cullum C. M. Zhang R. (2021). One-year aerobic exercise altered cerebral vasomotor reactivity in mild cognitive impairment[J]. J. Appl. Physiol. 131, 119–130. doi: 10.1152/japplphysiol.00158.2021, PMID: 34013755 PMC8325610

[ref62] Tsai C. L. Ukropec J. Ukropcová B. Pai M.-C. (2018). An acute bout of aerobic or strength exercise specifically modifies circulating exerkine levels and neurocognitive functions in elderly individuals with mild cognitive impairment[J]. Neuroimage Clinical 17, 272–284. doi: 10.1016/j.nicl.2017.10.028 29527475 PMC5842646

[ref63] Tsai C. L. Wang C. H. Pan C. Y. Chen F. C. Huang T. H. Chou F. Y. (2014). Executive function and endocrinological responses to acute resistance exercise[J]. Front. Behav. Neurosci. 8:262. doi: 10.3389/fnbeh.2014.00262, PMID: 25136300 PMC4117935

[ref64] Tsukamoto H. Suga T. Takenaka S. Takeuchi T. Tanaka D. Hamaoka T.. (2017). An acute bout of localized resistance exercise can rapidly improve inhibitory control[J]. PLoS One 12:e0184075. doi: 10.1371/journal.pone.0184075, PMID: 28877232 PMC5587287

[ref65] Tsukamoto H. Suga T. Takenaka S. Tanaka D. Takeuchi T. Hamaoka T.. (2016). Greater impact of acute high-intensity interval exercise on post-exercise executive function compared to moderate-intensity continuous exercise[J]. Physiol. Behav. 155, 224–230. doi: 10.1016/j.physbeh.2015.12.021, PMID: 26723268

[ref66] Urquhart E. L. Wanniarachchi H. I. Wang X.. (2019). Mapping cortical network effects of fatigue during a handgrip task by functional near-infrared spectroscopy in physically active and inactive subjects[J]. Neurophotonics 6:45011. doi: 10.1117/1.NPh.6.4.045011, PMID: 31853458 PMC6904890

[ref67] Uysal N. Kiray M. Sisman A. Camsari U. Gencoglu C. Baykara B.. (2015). Effects of voluntary and involuntary exercise on cognitive functions, and VEGF and BDNF levels in adolescent rats[J]. Biotech. Histochem. 90, 55–68. doi: 10.3109/10520295.2014.946968, PMID: 25203492

[ref68] Valer J. Daisuke T. Ippeita D. (2007). 10/20, 10/10, and 10/5 systems revisited: their validity as relative head-surface-based positioning systems [J]. NeuroImage 34:123. 10.1016/j.neuroimage.2006.09.024 17207640

[ref69] Vickers D. Vincent N. Medvedev A. (1996). The geometric structure, construction, and interpretation of path-following (trail-making) tests[J]. J. Clin. Psychol. 52, 651–661. doi: 10.1002/(SICI)1097-4679(199611)52:6<651::AID-JCLP7>3.0.CO;2-N, PMID: 8912108

[ref70] Weng T. B. Pierce G. L. Darling W. G. Voss M. W. (2015). Differential effects of acute exercise on distinct aspects of executive function[J]. Med. Sci. Sports Exerc. 47, 1460–1469. doi: 10.1249/MSS.0000000000000542, PMID: 25304335

[ref71] Yanagisawa H. Dan I. Tsuzuki D. Kato M. Okamoto M. Kyutoku Y.. (2010). Acute moderate exercise elicits increased dorsolateral prefrontal activation and improves cognitive performance with Stroop test[J]. NeuroImage 50, 1702–1710. doi: 10.1016/j.neuroimage.2009.12.023, PMID: 20006719

[ref72] Yang Y. Chen T. Shao M. Yan S. Yue G. H. Jiang C. (2020). Effects of tai chi Chuan on inhibitory control in elderly women: an fNIRS study[J]. Front. Hum. Neurosci. 13:476. doi: 10.3389/fnhum.2019.00476, PMID: 32038205 PMC6988574

[ref73] Yoon D. H. Kang D. Kim H. Kim J. S. Song H. S. Song W. (2017). Effect of elastic band-based high-speed power training on cognitive function, physical performance and muscle strength in older women with mild cognitive impairment[J]. Geriatr Gerontol Int 17, 765–772. doi: 10.1111/ggi.12784, PMID: 27396580

[ref74] Zhai S. Tanimura A. Graves S. M. Shen W. Surmeier D. J. (2018). Striatal synapses, circuits, and Parkinson's disease[J]. Curr. Opin. Neurobiol. 48, 9–16. doi: 10.1016/j.conb.2017.08.004, PMID: 28843800 PMC6022405

[ref75] Zhu Y. Zhong Q. Ji J. Ma J. Wu H. Gao Y.. (2020). Effects of aerobic dance on cognition in older adults with mild cognitive impairment: a systematic review and meta-analysis[J]. J. Alzheimers Dis. 74, 679–690. doi: 10.3233/JAD-190681, PMID: 32083578

